# You’re not alone: imagery rescripting for adolescents who self-harm

**DOI:** 10.3389/fpsyg.2024.1395603

**Published:** 2024-05-02

**Authors:** Elisa Schmied, Lisa Hack, Bernhard Connemann, Zrinka Sosic-Vasic, Julia Kroener

**Affiliations:** ^1^Christophsbad Goeppingen, Department of Applied Psychotherapy and Psychiatry, Göppingen, Germany; ^2^Medical Department, University of Ulm, Ulm, Germany

**Keywords:** non-suicidal self-injury, NSSI, imagery rescripting, adolescence, short-intervention, digital health intervention, psychotherapy, DHI

## Abstract

**Introduction:**

Non-suicidal self-injury (NSSI), which refers to the deliberate act of causing harm to one’s own body without the intent to commit suicide, occurs in 20% of youth. Interestingly, approximately 90% of individuals who engage in self-harm report intrusive mental imagery thereof shortly prior to the act of NSSI. Previous research has demonstrated that imagery rescripting (IR) is an effective technique to treat intrusive mental images and associated clinical symptoms, such as emotion dysregulation, in various psychiatric disorders. However, there is no research on IR for adolescents who self-harm. Therefore, the present study aims to investigate the efficacy and feasibility of a two-session short-intervention using IR to reduce NSSI and associated clinical symptoms in adolescents. The intervention was supported by an app-based digital health intervention (DHI).

**Methods:**

A single case series A-B design with three post-assessments (1 week, 1 month, and 3 months post-intervention) was implemented. Seven adolescents received two treatment sessions of IR, supported by a DHI between sessions. NSSI (SITBI), emotion regulation (ERQ), emotional distress (BDI-II, STAI-T), self-efficacy (WIRKALL_r), and treatment satisfaction (BIKEP) were evaluated.

**Results:**

There was an increase in adaptive emotion regulation strategies up to 3 months post-intervention. Furthermore, patients improved regarding their self-efficacy, depressiveness, anxiety, and NSSI symptomatology. The developed DHI was described as a helpful and supportive tool.

**Conclusion:**

The intervention has shown initial evidence to be feasible and beneficial for adolescents conducting NSSI. The DHI has demonstrated to be a valuable tool in the treatment of self-harming youth.

## 1 Introduction

Targeting non-suicidal self-injury in young people is a high priority, as it is a clinically relevant and prevalent activity that peaks during adolescence, with 20% of teenagers being affected (Plener et al., 2016). These adolescents face an increased likelihood of future NSSI as well as future acts of suicide (Groschwitz et al., 2015; Hawton et al., 2015). Yet, prior to the COVID-19 pandemic, approximately half of school-aged adolescents did not seek care following an incidence of self-harm (Rowe et al., 2014). This lack of help-seeking behavior can be ascribed to reasons such as stigma and self-stigma related to self-harm (Piccirillo et al., 2020), as well as a lack of knowledge regarding where to get support (Czyz et al., 2013).

Despite the pressing need for therapeutic support for adolescents who self-harm, accessible options are limited. However, a comprehensive review and meta-analysis conducted by Kothgassner et al. (2020) provided an initial evaluation of various treatments for adolescents who self-harm. Specifically, the meta-analysis aimed to examine the efficacy of existing treatments, such as Dialectical Behavior Therapy for Adolescents (DBT-A) and Mentalization-Based Treatment for Adolescents (MBT-A), in reducing self-harm, suicidal thoughts, and depressive symptoms. While examining research conducted over the past two decades, the meta-analysis found that the effects of treatment interventions compared to active controls were generally small in magnitude. The sole therapeutic approach that has demonstrated encouraging outcomes for adolescents who self-injure is DBT-A (Kothgassner et al., 2020, 2021). However, it necessitates the involvement of numerous therapists and a substantial time commitment to the therapeutic process. Therefore, it may not be broadly applicable and available, necessitating short-interventions providing targeted and low-threshold accessibility.

Recent findings indicate that dysfunctional mental imagery may contribute to self-harm behavior (May et al., 2015). Interestingly, 90% of individuals who engage in NSSI experience mental images prior to engaging in such behavior (McEvoy et al., 2017). Mental imagery is a multisensory process that is highly emotionally evocative above pure verbal processing (Nock et al., 2006). Research findings indicate that individuals utilize these images as a coping mechanism for managing aversive emotions such as feelings of loneliness, or as a form of self-punishment (Holmes and Mathews, 2005; Schaitz et al., 2020). In addition, imagining future behavior can have a motivational impact and can influence the likelihood of executing this behavior (Holmes and Mathews, 2010). This assertion is substantiated by neurofunctional research, which suggests that mental imagery of a specific action and subsequently performing that action is facilitated by the activation of overlapping brain areas (Libby et al., 2007). Individuals who self-harm often report vivid mental imagery prior to engaging in NSSI (Knäuper et al., 2009). Across psychopathology, mental imagery can act as an emotional and motivational ‘amplifier’ (Hasking et al., 2018). Before engaging in non-suicidal self-injury (NSSI), the use of imagery can increase negative emotions, leading to a desire to self-harm. Additionally, imagery can also encapsulate the expected relief that is associated with self-harm, consequently inheriting both an affective and motivational component within NSSI. Recent research indicates that imagery may play a significant role in the onset of non-suicidal self-injury (NSSI). Knäuper et al. (2009) have observed the presence of highly emotional imagery preceding NSSI, which could potentially influence both the emotional cascade and dysregulation that contribute to NSSI, as well as the subsequent behavioral manifestations.

Innovative psychological treatments that utilize new mechanistic approaches provide alternative treatments to target and modify the underlying processes that sustain symptoms (Holmes et al., 2018). The utilization of IR is a widely recognized cognitive approach that has been commonly implemented in therapeutic protocols for several psychiatric disorders (for reviews, see Kip et al., 2023; Kroener et al., 2023). Thus far, scientific studies have been conducted to explore the efficacy of interventions utilizing imagery as a means to address NSSI. A recent study conducted by Schaitz et al. (2020) developed and implemented a brief therapeutic intervention consisting of two sessions IR. This intervention aimed to address intrusive imagery that is linked to emotionally dysregulated behavior (EDB) such as NSSI, binge eating, or high-risk sexual contact. The intervention involved the identification, analysis, and modification (referred to as “rescripting”) of these images, which were subsequently rehearsed on a daily basis at home. The objective of the IR was to diminish the adverse emotions and dysfunctional cognitive processes linked to EDB through the substitution of unhealthy and dysfunctional mental images with more adaptive content. Sosic-Vasic et al. (2024) expanded on the initial findings by Schaitz et al. (2020) by conducting a randomized controlled trial (RCT) investigating the same mechanisms. Both studies revealed that mechanisms such as EDB, emotion dysregulation, or depressiveness can be targeted and improved using two sessions of IR. Furthermore, a study conducted by Simplicio et al. (2020) introduced a Functional Imagery Training (FIT) paradigm that specifically focuses on addressing self-harm behaviors. This protocol utilizes motivational imagery techniques as a means to facilitate behavioral modifications. In contrast to the studies conducted by Schaitz et al. (2020) and Sosic-Vasic et al. (2024), FIT does not specifically address the pre-existing negative mental images related to self-harm. Instead, individuals are taught to use motivational images at times when they feel the urge to self-harm. Furthermore, FIT was combined with a smartphone app for young people. Within the application, patients were directed to utilize guided imagery audio recordings along with a feeling-thermostat and a recommendation for activities that may be beneficial depending on their emotional state. The benefit of the app is to promote self-management skills. The aim was to engage more young people by leveraging their familiarity with phone apps (Ofcom, 2015). Taking the previous findings together, the studies conducted by Schaitz et al. (2020) and Sosic-Vasic et al. (2024) have demonstrated the feasibility of rescripting aversive imagery linked to NSSI in adults who self-harm, whereas the study implemented by Simplicio et al. (2020) has shown that generating positive imagery can reduce self-harm in adolescents. Both of these studies substantiate the efficacy of imagery-based approaches, particularly in the context of modifying aversive imagery linked to NSSI. These approaches have proven to be promising in reducing NSSI behaviors among adolescents, therefore serving as the foundational rationale for the implementation of our intervention.

Interestingly, research has started to provide blended care combining regular psychotherapeutic approaches with digital health interventions (DHI) with increased popularity (Lee et al., 2018; Simplicio et al., 2020), especially among young people who heavily rely on the usage of smartphones (Torous et al., 2014; Larsen et al., 2016). These digital tools can support self-management skills and provide access to mental health care, reducing barriers and stigma (Torous et al., 2014). Blended interventions combining app usage and in-person contact with a therapist are preferred by young people (Hollis et al., 2017). However, there are only a few evidence-based apps specifically targeting adolescent self-harm behavior (Simplicio et al., 2020). FIT used an imagery-based approach but failed to directly address NSSI related aversive imagery, while the studies conducted on EDB in adult patients diagnosed with BPD (Schaitz et al., 2020; Sosic-Vasic et al., 2024) targeted imagery processes, but failed to implement DHIs. Therefore, the present study shall combine the two previous approaches by conducting IR supported by a digital app that should promote the transfer into everyday life. The present study focuses on NSSI related aversive images and attempts to enhance treatment efficacy over time through the use of a digital self-management tool that was developed in collaboration with adolescents. The app entails a translation and extension of the FIT-app (Simplicio et al., 2020). However, unlike the FIT-app, the present software solely emphasizes mental imagery: Patients can choose between two options (a) accessing a range of pre-existing mental imagery audios within the app, or (b) listening to specific audios recorded during their two in-person sessions with their therapist. While the FIT-app is only available for IOS, our app is available for IOS and Android devices. Ultimately, the design is enhanced to be more appealing to the specific age demographic being targeted.

Concluding out of previous scientific evidence, the current study aims to investigate the feasibility and efficacy of a two-session short-intervention using IR. The intervention will be supported by an app-based DHI to reduce NSSI, and increase several aspects of emotion dysregulation and self-efficacy in adolescents who self-harm. The primary objective of this Phase I feasibility study is to establish the framework for subsequent Phase II trials of a novel intervention for NSSI. We believe that this intervention is highly sought after due to its brevity and its potential adaptability and scalability.

## 2 Materials and methods

### 2.1 Design

A single case series A-B design was implemented including two follow-up assessments. This design was chosen to assess the feasibility of implementing IR within adolescents conducting NSSI. The study was conducted as a one-arm trial, with assessments at four distinct time-points: pre-intervention (T0), post-intervention (1 week after treatment termination, T1), and two follow-up assessments [1 month (FU1), and 3 months (FU2) after treatment termination]. One independent assessor conducted diagnostic assessments and evaluations for symptom improvement.

### 2.2 Patients

Seven patients fulfilling clinical diagnosis of NSSI (DSM-5; Association, American Psychiatric, 2013; R45.88/Z91.5; Remschmidt et al., 2017) were recruited between January 2022 and January 2023. The entire sample reported German as their native language. None of the patients received psychotherapy while attending the study. The patients were recruited via the University Clinic of Ulm (Department of Psychiatry and Psychotherapy III), from waiting lists of psychotherapists, youth welfare offices in Ulm, and through social media. Exclusion criteria were: (a) acute suicidality, (b) current alcohol abuse, dependence, or other substance-related disorders, (c) a history of psychotic or bipolar disorders. Inclusion criteria were meeting diagnostic criteria of NSSI from the DSM-5 (SITBI; Nock et al., 2006), reporting recurrent images of NSSI, and age between 13 and 24. Patient characteristics are shown in Table 1.

**Table 1:** Patient characteristics.

	Patient 1	Patient 2	Patient 3	Patient 4	Patient 5	Patient 6	Patient 7
Age	15	16	14	24	18	15	14
Sex	Female	Female	Female	Female	Female	Female	Female
Education	Currently in high school	Currently in high school	Currently in middle school	Bachelor degree	Currently in high school	Currently in middle school	Currently in middle school
Age first NSSI-behavior	13	10	11	15	13	12	14
Age first NSSI-thoughts	13	10	12	13	13	12	13
Comorbid disorders	Depression	Depression, Anorexia	Depression, PTSD, Bulimia	GAD	None	Depression, social phobia, GAD	Depression, panic-disorder, agoraphobia, social phobia
Psychopharmaca	No	No	No	No	No	No	No
No. of NSSI-behavior past year	50	5	100	120	70	150	30
No. of NSSI-thoughts past year	50	18	170	120	150	70	300
Reasons for NSSI	Self-punishment	Started with sexual abuse	Maybe because of my past (trauma)	To regulate emotions and stress	Because I can get rid of my thoughts	To get rid of tension and to deal with sadness	Because it is the only way
Type of NSSI	Cutting, self-biting, scratch skin	Cutting, self-hitting, manipulation of a wound, self-burning, self-biting, scratch skin, chafing the skin until it bleeds	Cutting, scratch skin, chafing the skin until it bleeds	Manipulation of a wound, self-biting, skin-picking until it bleeds	Cutting, manipulation of a wound, scratch skin	Cutting, self-hitting, manipulation of a wound, self-burning, self-biting, skin-picking until it bleeds, scratch skin, chafing the skin until it bleeds	Cutting, self-hitting, pull out hair, manipulation of a wound, objects under nails, self-biting, scratch skin, chafing the skin until it bleeds
Medical wound management	No	No	No	No	No	No	No
Psychotherapy history	No	Yes	Yes	No	No	No	Yes

No, number; NSSI, Non-suicidal self-injury; PTSD, Posttraumatic stress disorder; GAD, Generalized anxiety disorder.

After the initial screening, patients were scheduled for a diagnostic session (T0), during which the diagnosis of NSSI was established using the SITBI (Nock et al., 2006). Moreover, the Mini-International Neuropsychiatric Interview (M.I.N.I.) for DSM-4 and ICD-10 (Sheehan et al., 1998) was implemented to evaluate comorbid Axis-I diagnoses (see Table 1 for comorbid disorders). Additionally, NSSI related mental imagery was assessed using an adapted version of the Imagery Interview (Hackmann et al., 2000). Lastly, patients were screened for inclusion-, and exclusion criteria. Thereinafter, eligible patients provided written informed consent and were instructed to complete standardized questionnaires.

To avoid concurrent treatment effects, patients who were recruited during their inpatient treatment were included in the study after treatment completion. Some of the patients never had any contact with the health system before participating in the study due to feared stigmatization. Therefore, none of the included patients received psychotherapeutic treatment during study participation (T0-FU2).

### 2.3 Intervention

The intervention consisted of two individual sessions of IR, with one session per week for two consecutive weeks. During each IR session, patients were instructed to identify and describe a distressing mental image related to NSSI. This image was then transformed into a more positive and pleasant image. Specifically, the therapist helped the patient to identify the point of no return (i.e., the point within the image, where the subsequent self-injurious behavior became inevitable) and guided the patient to imagine a different outcome including adaptive coping behaviors. Each session lasted about 100 min.

Between the two treatment sessions, patients utilized an app, which was developed within the current study based on the FIT-app (Simplicio et al., 2020). Within this app, patients listened to audio recordings of the IR sessions, completed diary cards, and provided feedback afterwards. Additionally, the app included psychoeducational content on NSSI and emotions, as well as other guided imagery audios similar to the FIT-app.

#### 2.3.1 First session and homework

At the beginning of the session patients were provided with information about the possible benefits of working with intrusive images. Furthermore, the concept of IR was introduced as follows:

*During our diagnostic interview, you described experiencing images of self-harm during times of heightened stress. While imagining the latter behavior, you may notice that your stress level decreases. Subsequently, your brain creates a connection between these self-harming images and the experience of reduced tension. This connection is responsible for an increased likelihood of actually engaging in NSSI. Today, we will work on these images using a technique called IR. Doing so, you will describe one of these images using all senses, such as what you see, hear, and smell. We will also explore the associated thoughts and feelings. Your task is to be the narrator of the described image. My task will be to create a written story of your description. IR can be best explained by the fairy tale of Hansel and Gretel. In this story, Hansel and Gretel are abandoned, which is very unpleasant for them. Feelings such as fear, sadness, or anger arise. Eventually, Hansel and Gretel approach the witch’s house, and stand in front of the house, only to be captured by the witch. Once the witch opens the door, we know that something bad is going to happen. This is the point of no return. If we used IR to re-write the story, Hansel and Gretel would not enter the witch’s house but take a different path, which, for example, leads them back home safely. This means that during the IR, we will formulate an alternative ending resulting in a benign outcome. We will use the same method with one of your images of self-harm. Initially, you may experience unpleasant feelings, but we will work together to create an alternative ending to your story. Do you have any questions about this? Please use “I” statements and present tense. You will now describe your image until your point of no return. Afterwards, I will provide you with information about the rescripting part, followed by working together on the formulation of an alternative ending. Are you ready?*

After the IR intervention, the therapist and patient worked jointly to create a written script, which captured the newly developed image. Thereinafter, the written script was recorded using the app. Additionally, the patient received an introduction into the app. Lastly, the patient was instructed to imagine the rescripted image daily for the following week.

#### 2.3.2 Phone call

After each therapeutic session, the therapist followed up with the respective patient by phone call within 1 week post-treatment, in order to assess occurring difficulties while using the app (approx. 15 min).

#### 2.3.3 Second session

At the beginning of the second session, the therapist inquired about any difficulties encountered while completing the assigned homework during the previous week. Additionally, negative effects experienced after the first IR session were evaluated, and any adjustments for the second IR session were clarified. Additionally, any open questions were addressed. Afterward, another distressing mental image related to self-harm was chosen and rescripted based on the previous IR session. Toward the end of the second session, the therapist addressed any remaining questions, sought feedback from the patient regarding the intervention, recommended daily practice of the rescripted images, and encouraged the patient to apply the learned technique to any future distressing mental images of self-harm.

#### 2.3.4 Post-diagnostics and follow-up session

The post-diagnostic sessions took place 1 week after the last IR session (T1). The follow-up assessments took place 1 month and 3 months after the last treatment session (FU). During all assessment time-points, patients were asked about their experiences using the app. Furthermore, questionnaires were completed during each assessment.

### 2.4 Instruments

Imagery Interview (Hackmann et al., 2000). The structured Imagery interview uses standardized questions. Initially, the therapist explains mental images using an example and asks general questions about intrusive images. Thereinafter, the patient is instructed to imagine an NSSI related image and answer questions regarding this image. Afterwards, the therapist and the patient discuss the image’s content, meaning, emotions, and contributing factors. Then, associated thoughts are explored. The patient also answers questions about their experience with the image.

M.I.N.I (Sheehan et al., 1998). To assess possible comorbid psychiatric disorders, the “M.I.N.I. International Neuropsychiatric Interview,” which allows for a structured diagnostic approach, was conducted.

### 2.5 Outcomes

Parts of the structured “Self-Injurious Thoughts and Behaviors Interview” (SITBI; Nock et al., 2006) were used to assess non-suicidal self-injury. The complete questionnaire consists of 169 items divided into five areas: Onset, frequency, methods, functions, and social influences.

We defined NSSI according to the DSM-5 criteria (Association, American Psychiatric, 2013). Inclusion criteria according the DSM-5 are at least five or more self-harming incidents during the past 12 month, Moreover, NSSI-incidents are preceded by interpersonal problems, negative emotional states, or frequent thoughts about NSSI. Within the SITBI questionnaire, item 147 asks about the number of injuries in the past year, and item 50 asks about the type of injury.

Emotion Regulation Questionnaire (ERQ; German Version; Abler and Kessler, 2009). The ERQ is a self-report questionnaire consisting of 10 items that measure two separate strategies for regulating one’s positive and negative emotions: cognitive reappraisal and expressive suppression. Each item can be rated on a 7-point Likert scale, with 1 indicating strong disagreement and 7 indicating strong agreement. The internal consistency ranges from alpha = 0.74 to alpha = 0.76 (Abler and Kessler, 2009).

General Self-Efficacy Expectancy Scale (WIRKALL_r; German Version; Schwarzer, 1999). This scale assesses general self-efficacy at the hand of 10 items that are answered on a four-point scale. The WIRKALL_r has high internal consistency of 0.86 (Schwarzer, 1999).

The Beck Depression Inventory (BDI-II; German Version; Hautzinger et al., 2006) is a self-report measure consisting of 21 items that assess symptoms of depression experienced in the past 2 weeks, including today. Each item can be rated on a 4-point Likert scale, with a score of 0 indicating the absence of symptoms and a score of 3 indicating severe symptomatology. The questionnaire demonstrates high internal consistency, with a Cronbach’s alpha of 0.90 (Hautzinger et al., 2006).

The State–Trait Anxiety Inventory-Trait Version (STAI-T; Grimm, 2009) focuses on anxiety as a trait, i.e., anxiety as a relatively enduring personality trait (trait anxiety), which refers to individual differences in the tendency to respond to feared stimuli. The scale contains 20 items and has an internal consistency of alpha = 0.90 (Grimm, 2009).

#### 2.5.1 Acceptance

The Bielefeld Client Experience Questionnaire (BIKEB; Höger and Eckert, 1997) was used to assess acceptance of the intervention. The BIKEB consists of 6 scales (getting along with the therapist, coming to terms with oneself, experiencing change, experiencing security and confidence, reassurance, physical relaxation vs. exhaustion) with 4 items each, which have a Cronbach’s alpha between 0.69 and 0.83 (Höger and Eckert, 1997). Additionally, a sum score can be built across all subscales demonstrating overall acceptance and treatment satisfaction. Within the present study we focused on 3 subscales: getting along with the therapist, experiencing change and experiencing security and confidence.

### 2.6 Statistical analysis

According to the implemented single case series design, data was visually inspected per patient in order to determine treatment effects. This procedure allows for the evaluation of each individual’s change over time, as well as the assessment of each patient’s range and stability of change. However, the mere evaluation of descriptive data might result in Type I error. Henceforth, changes in outcome measures were analyzed using percentage values. Treatment response (defined as a 30% reduction in NSSI behavior) was evaluated for each patient. Additionally, reliable change as measured by the RCI (Jacobson and Truax, 1991) was assessed for each patient using standard deviations of previous studies including adolescents conducting NSSI (James et al., 2008; Esposito et al., 2023) on clinical outcome measures assessing NSSI behavior and depressiveness (BDI-II). A RCI ≥ 1.96 is indicative 3for a significant change.

Moreover, paired sample t-tests across all measurement time-points (i.e., T0, T1, FU1, FU2) were implemented to assess changes from pre-, to post-treatment and follow-up for the overall group. Pre-, post- and follow-up effect sizes were calculated using Cohen’s d (Field, 2013).

## 3 Results

A description of intrusive images, associated emotions and rescripts, as evaluated during treatment, as well as the app usage can be found in Table 2.

**Table 2:** Rescripted images and associated emotions, app usage and app feedback.

	Patient 1	Patient 2	Patient 3	Patient 4	Patient 5	Patient 6	Patient 7
Imagination in session 1	Cutting in own room	Cutting in own room	Cutting in room	Starting to bite check in room	Cutting in room	Cutting in bathroom	Cutting in bathroom
Main emotions during imagery	Loneliness, sadness	Loneliness, exhaustion, sadness, tension	Loneliness, anxiety, anger, tension	Loneliness, tension, anxiety, excitement	Loneliness, tension, anxiety	Anxiety, tension, anger, sadness	Loneliness, feeling nothing, numb, dissociation
Rescript of NSSI session 1	Play a gameboard with brother	Doing acrobatics	Talking with best friend over the phone	Music and dancing with boyfriend and friends	Riding a horse	Playing with baby brother	Talking with boyfriend
Listening to audios session 1	7	6	6	9	4	6	3
Listening to audios session 2	9	4	5	6	3	3	1
Feedback app	Good app, it would be nice to include a diary and a daily planner	Good app, no further comments	Good app, helps a lot	The reminder function was great. The length of the audios was easy to integrate into everyday life. I some-times found the more negative part difficult, but the positive part helped me a lot. It has helped me a lot, after two weeks. I just feel that my Audios no longer quite match how I feel, which is good, because I have clearly moved on	I found the app really great to use and I think it’s good that you can set reminders via the app	More options to select feelings after listening to your own and provided audios	Good idea, but did not help me that much

NSSI, Non-suicidal self-injury.

Table 3 presents scores for all patients, measurements and measurement time-points. Looking at NSSI behavior (SITBI), there was a non-significant 15% reduction of self-harming behavior 1 week after treatment termination, t (6) = 0.36, *p* > 0.05, *d* = 0.13. However, there was a significant 48% reduction in NSSI behavior 1 month after treatment termination, t(6) = 2.46, *p* ≤ 0.05, *d* = 0.93, and a significant 79% reduction of NSSI behavior 3 months after treatment termination for the group as a whole, t(6) = 1.97, *p* ≤ 0.05, *d* = 0.75, demonstrating that further gains were made during the follow-up period. Six out of seven patients met criteria for being treatment responders (≥ 30% reduction in NSSI behavior at follow-up) whereby 5 of 7 patients (1, 2, 3, 6, 7) revealed RCIs greater than 1.96 at both follow-up time-points, indicating significant changes in NSSI behavior. One patient (patient 4) was a non-responder. This patient showed a 40% increase in NSSI behavior at FU1, and a 100% increase at FU2.

**Table 3:** Outcome measures and symptom reduction across rescripting therapy.

		Patient 1	Patient 2	Patient 3	Patient 4	Patient 5	Patient 6	Patient 7	Mean (*SD*)	Effect size *d*
NSSI-behavior	T0	10	3	3	5	2	11	20	7.71 (6.5)	
	T1	0	0	0	1	3	4	12	6.57 (6.68)	0.13
	FU 1	1	0	0	7	2	3	15	4 (5.4)	0.93**
	FU 2	0	0	0	10	0	3	0	1.86 (3.76)	0.75**
% reduction on NSSI-behavior	T0-T1	100%	100%	100%	80%	50% (increase)	64%	40%	100%	
	T0-FU1	90%	100%	100%	40% (increase)	0%	73%	25%	90%	
	T0-FU2	100%	100%	100%	100% (increase)	100%	73%	100%	100%	
NSSI-thoughts	T0	10	4	10	15	11	12	30	13.14 (8.13)	
	T1	7	1	19	6	25	15	28	3.71 (0,.5)	1.24**
	FU1	0	1	0	7	12	6	25	7.29 (8.98)	1.47**
	FU2	0	4	0	15	10	12	30	10.14 (10.52)	0.63
BDI-II	T0	39	53	70	34	55	62	74	55.29 (14.92)	
	T1	27	48	36	28	58	56	64	45.29 (15.02)	0.86
	FU1	32	47	32	25	42	47	70	42.14 (14.85)	1.13
	FU2	35	43	21	27	31	43	60	37.14 (12.89)	1.19
% reduction on BDI-II score	T0-T1	31%	9%	49%	18%	5% (increase)	10%	14%	31%	
	T0-FU1	18%	11%	54%	26%	24%	24%	5%	18%	
	T0-FU2	10%	19%	70%	21%	44%	31%	19%	10%	
ERQ reappraisal	T0	28	24	14	19	20	20	11	18.86 (5.84)	
	T1	28	28	26	27	21	16	16	24.43 (4.43)	1.28***
	FU1	34	27	24	30	22	16	16	27 (4.7)	2.80****
	FU2	36	30	29	30	23	19	19	27.71 (6.4)	2.38****
ERQ suppression	T0	21	14	28	14	20	16	19	19.43 (4.76)	
	T1	19	16	20	8	21	25	13	16.14 (4.53)	0.87**
	FU1	14	12	20	7	28	27	22	16.14 (5.61)	0.87**
	FU2	14	16	19	8	31	20	18	16.71 (4.75)	0.58
STAI-T	T0	43	60	70	54	62	73	76	62.57 (11.6)	
	T1	35	49	58	44	70	69	70	56.42 (14.13)	0.90**
	FU1	34	50	50	36	63	59	63	50.71 (12.02)	1.72***
	FU2	26	52	34	42	42	57	61	44.85 (12.58)	1.99****
WIRKALL_r	T0	21	28	16	24	24	15	10	19.7 (6.29)	
	T1	21	17	18	26	22	17	20	20.14 (3.23)	0.43
	FU1	25	29	23	29	24	21	22	24.71 (3.2)	1.25***
	FU2	24	29	26	29	31	18	20	25.28 (4.89)	1.57***

SD, Standard Deviation; T0, pre-intervention, T1, post-intervention, FU1, 1-month follow-up, FU2, 3-months follow-up; NSSI, Non-suicidal self-injury; ERQ, Emotion Regulation Questionnaire; BDI-II, Becks Depression Inventory; STAI-T, The State–Trait Anxiety Inventory-Trait Version; WIRKALL_r, General Self-Efficacy Expectancy Scale. *Mean total scores and standard deviations for NSSI thoughts and NSSI behavior, ERQ, STAI-T, WIRKALL_r, and BDI-II for all participants at T0, T1, FU1 and FU2. Effect sizes are measured using Cohen’s d and were calculated using the pre assessment scores as baseline comparison. **p ≤ 0.05, ***p ≤ 0.01, ****p ≤ 0.001.

**Figure 1:**
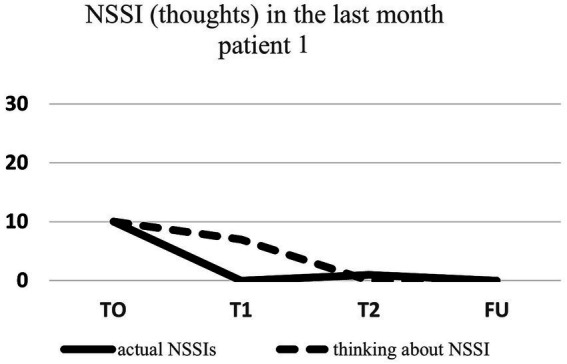
Individual NSSI behavior and NSSI thoughts over the course of the rescripting therapy.

**Figure 2:**
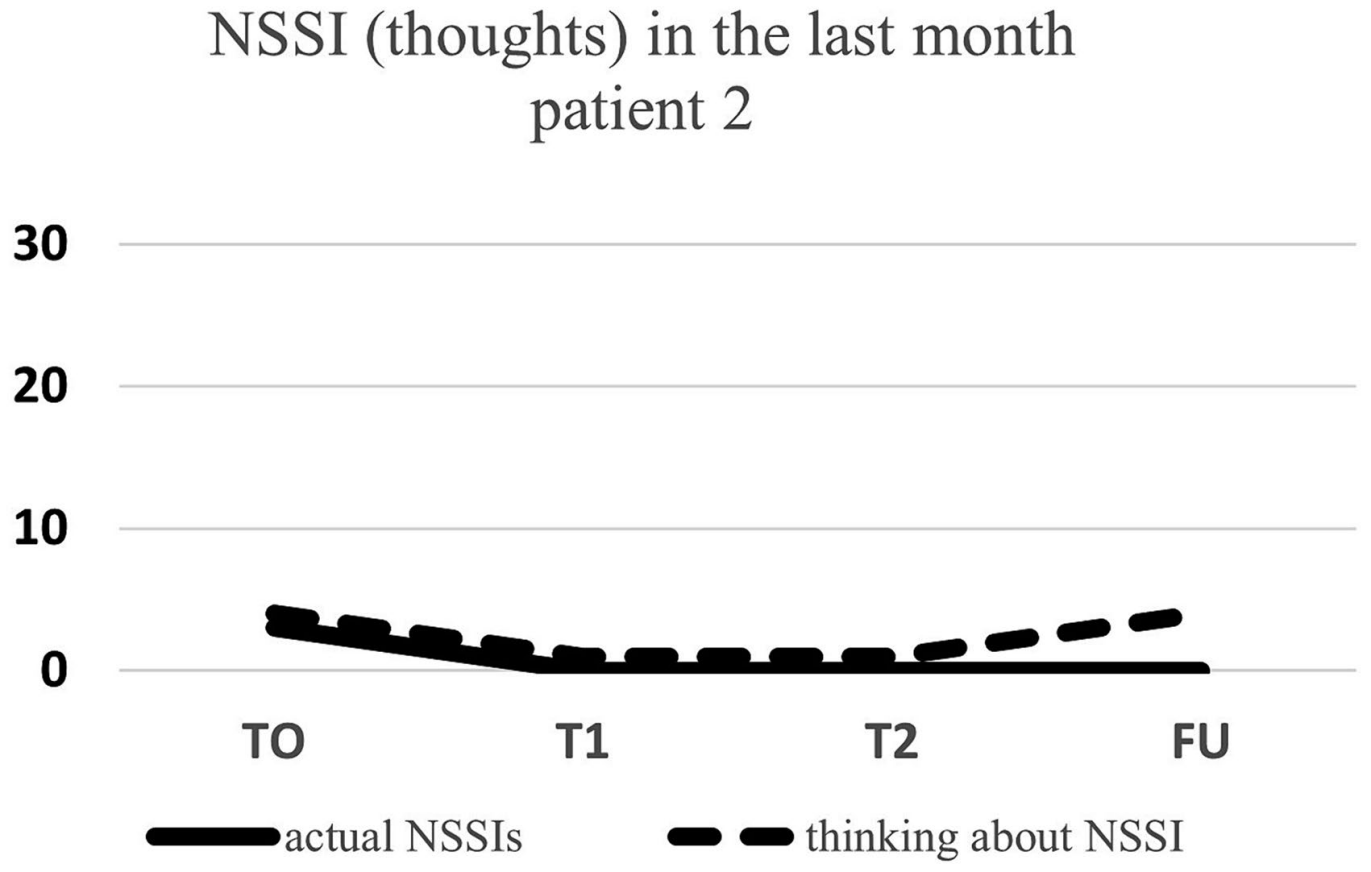
Individual NSSI behavior and NSSI thoughts over the course of the rescripting therapy.

**Figure 3:**
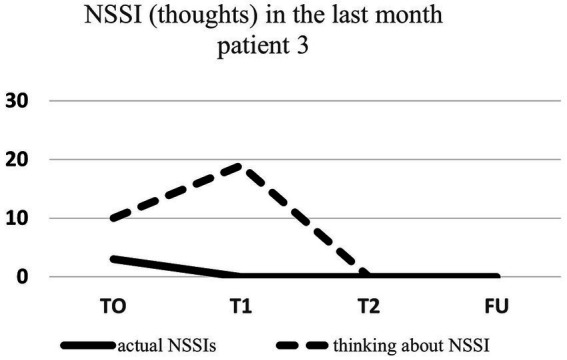
Individual NSSI behavior and NSSI thoughts over the course of the rescripting therapy.

**Figure 4:**
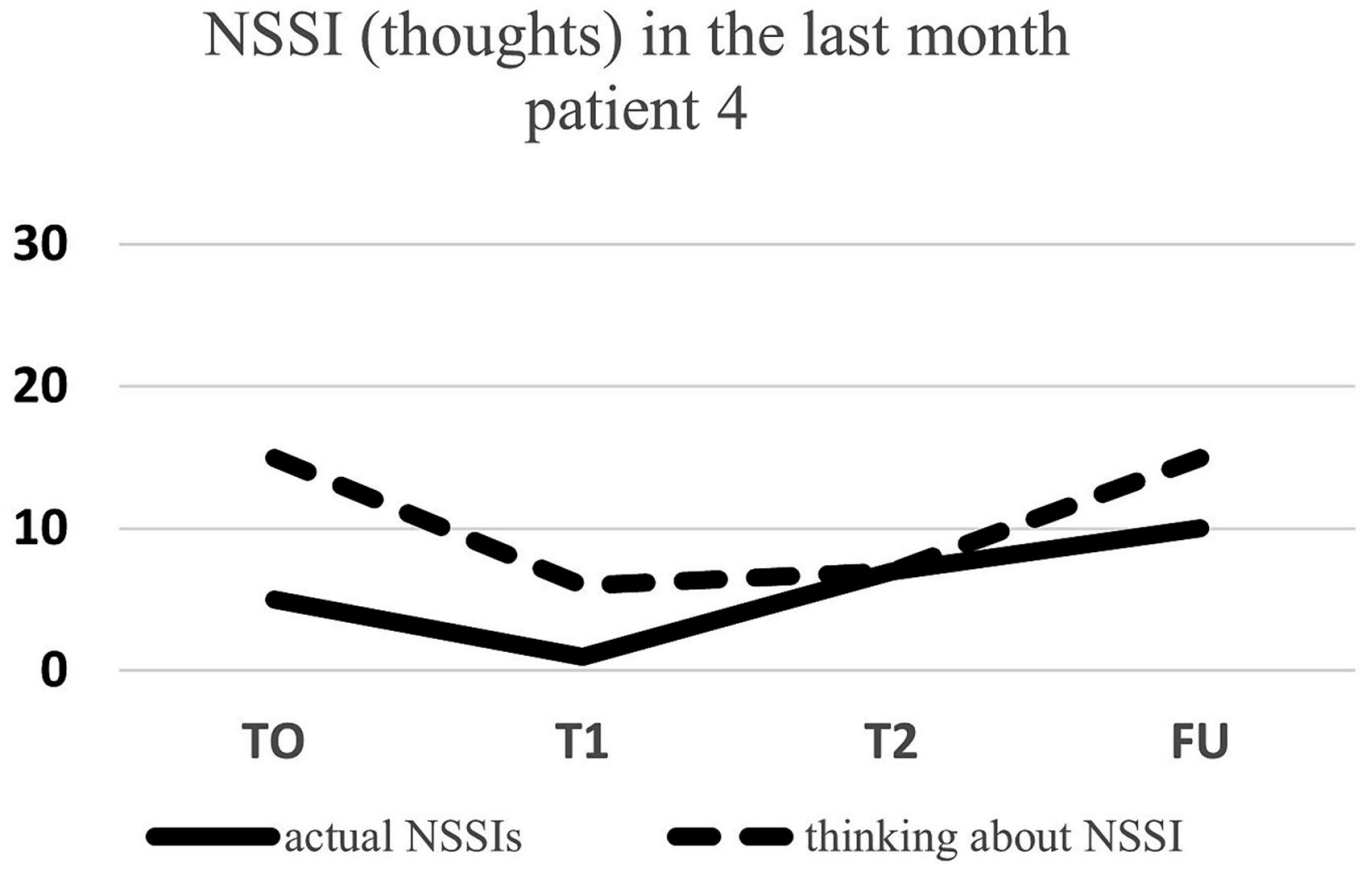
Individual NSSI behavior and NSSI thoughts over the course of the rescripting therapy.

**Figure 5:**
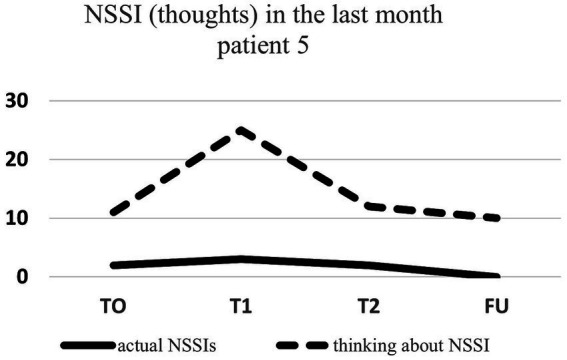
Individual NSSI behavior and NSSI thoughts over the course of the rescripting therapy.

**Figure 6:**
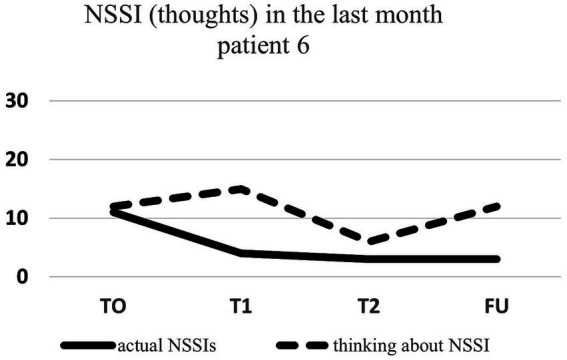
Individual NSSI behavior and NSSI thoughts over the course of the rescripting therapy.

**Figure 7:**
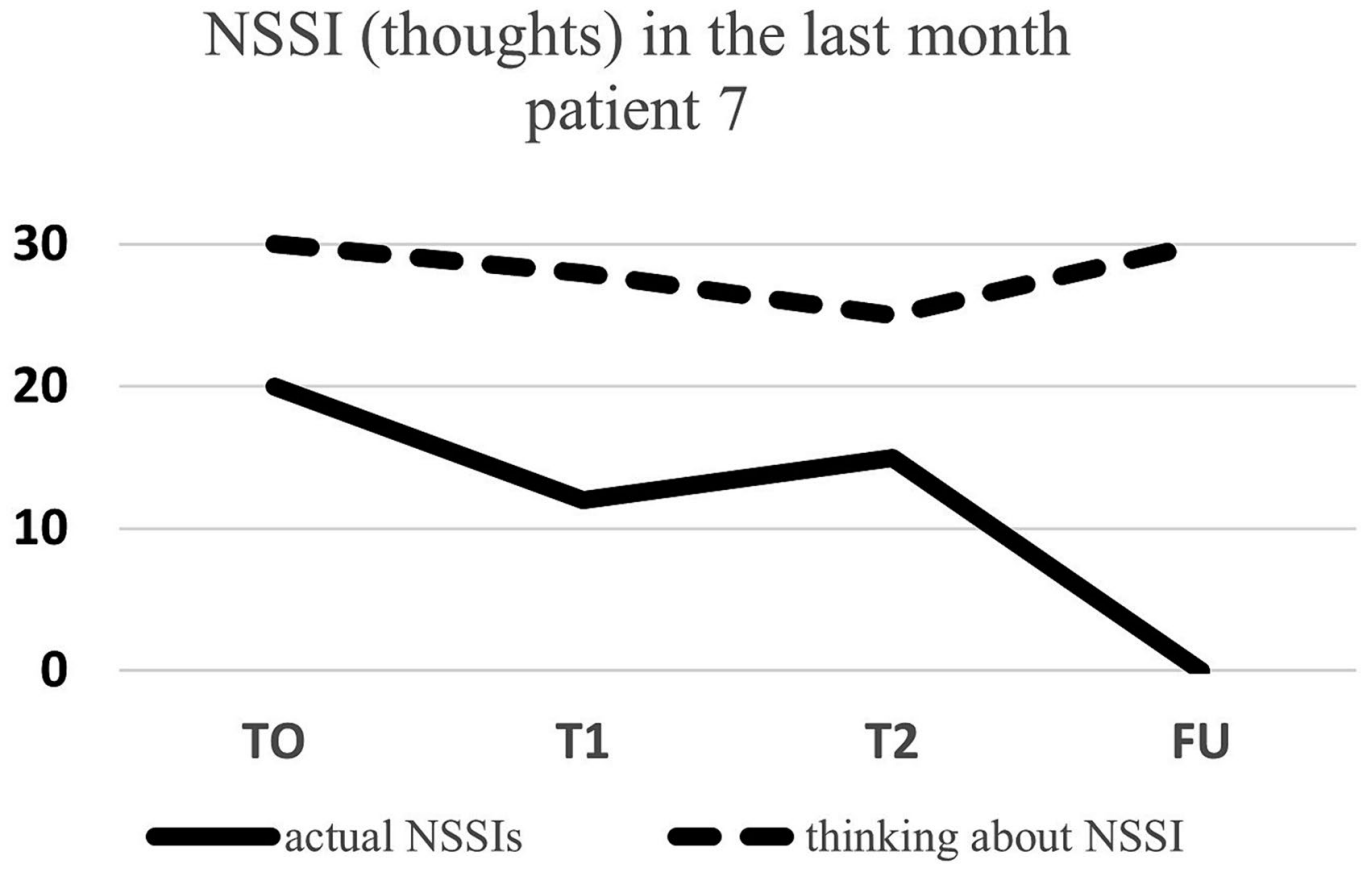
Individual NSSI behavior and NSSI thoughts over the course of the rescripting therapy.

Regarding NSSI related thoughts (SITBI), there was a significant 72% reduction in thoughts about NSSI 1 week after the intervention, t(6) = 3.27, *p* ≤ 0.01, *d* = 1.24, as well as a significant 45% reduction in the latter thoughts 1 month after the intervention, t(6) = 3.90, *p* ≤ 0.01, *d* = 1.47. Furthermore, there was a trend 23% reduction in NSSI thoughts 3 months after the intervention for the group as a whole, t(6) = 1.66, *p* = 0.07, *d* = 0.63. Changes in NSSI behavior and NSSI thoughts for each patient are shown in Figures 1–7.

With respect to depressive symptoms as assessed by the BDI-II, there was a significant 18% decrease in depressiveness for the group as a whole 1 week after treatment termination, t(6) = 2.28, *p* ≤ 0.05, *d* = 0.86, as well as a 24% reduction in depressive symptoms 1 month, t(6) = 2.99, *p* ≤ 0.01, *d* = 1.13, and a 33% decrease in depressiveness, t(6) = 3.14, *p* ≤ 0.01, *d* = 1.19, 3 months after treatment termination respectively, demonstrating that further gains were made during the follow-up period. Five out of seven patients (patient 2, 3, 5, 6, 7) revealed RCIs higher than 1.96, indicating clinically significant changes in depressive symptoms at 3 months follow-up. The remaining two patients (patient 1 & 4) did not display clinical change according to the RCI at 3 months follow-up, however, both displayed a 10% (patient 1) and 21% (patient 4) reduction in depressive symptoms as compared to the baseline measurement time-point.

### 3.1 Changes in emotion regulation, trait anxiety, and self-efficacy

Looking at emotion regulation, there was a significant 23% increase regarding the adaptive emotion regulation strategy reappraisal (ERQ) 1 week after treatment termination, t (6) = −3.39, *p* ≤ 0.01, *d* = 1.28, a 30% increase 1 month after treatment termination, t (6) = −7.4, *p* ≤ 0.001, *d* = 2.80, as well as a 32% increase 3 months after treatment termination t (6) = −6.31, *p* ≤ 0.001, *d* = 2.38, which displays that further gains were achieved during the follow-up period. For the maladaptive emotion regulation strategy suppression (ERQ), there was a significant 17% decrease 1 week after treatment termination t (6) = 2.30, *p* ≤ 0.05, *d* = 0.87, as well as a significant 17% decrease 1 month after treatment termination, t (6) = 1.95, *p* ≤ 0.05, *d* = 0.74. At 3 months follow-up, there was a trend 14% decrease in suppression, t (6) = 1.55, *p* = 0.09, *d* = 0.58.

Regarding general anxiety (STAI-T), there was a significant 10% decrease from baseline to post-treatment, t (6) = 2.38, *p* ≤ 0.05, *d* = 0.90. Furthermore, state anxiety significantly decreased about 19% 1 months after treatment termination, t (6) = 4.54, *p* ≤ 0.01, *d* = 1.72, and about 28% 3 months after treatment termination t (6) = 5.25, *p* ≤ 0.001, *d* = 1.99, demonstrating that further gains in symptom improvement were achieved during the follow-up period.

With respect to self-efficacy (WIRKALL_r), no significant change was achieved 1 week post-intervention as compared to baseline, t(6) = −0.18, *p* = 0.43. However, there was a significant 25% increase in self-efficacy 1 month after treatment termination, t(6) = −3.31, *p* ≤ 0.01, *d* = 1.25, as well as a significant 28% increase 3 months after treatment termination t (6) = −4.15, *p* ≤ 0.01, *d* = 1.57, showing increasing gains across the follow-up period.

### 3.2 Feasibility and acceptability

**Figure 8:**
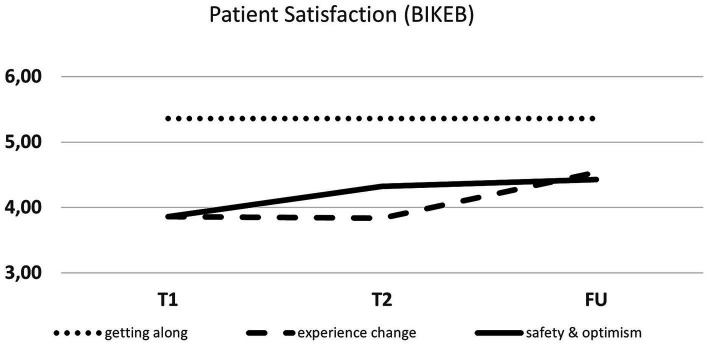
Patient satisfaction (BIKEB) across the rescripting therapy.

Recruitment procedures were successful. The target of recruiting seven adolescent patients with an NSSI diagnosis was achieved within 1 year. All included patients completed all IR treatment sessions as well as assessment time-points. No adjustments had to be made to the study protocol. Furthermore, treatment acceptability (BIKEB) was high for all included patients, ranging between 3.28 and 5.16 points (*M* = 4.22, *SD* = 0.66; see Figure 8 for a detailed report). Specifically, patients reported that the therapy they received helped them develop new perspectives and that they now approach the future with greater confidence after receiving IR treatment. Moreover, patients said that they acquired the ability to associate their behavior with their emotional responses. Finally, the utilization of the application was found to be substantial, as indicated by an average of 5.86 times of listening to the audio scripts between the first and second intervention session, and 4.43 times between the second treatment session and the post-assessment.

Overall, patient response pertaining to the application was largely positive, with individuals expressing that the app, audio-scripts, and reminders proved to be quite helpful. One patient (patient 1) suggested including a daily planner within the app. Lastly, one patient (patient 7) stated that the app was not very helpful without further specifications.

## 4 Discussion

The aim of the present study was to investigate the feasibility and efficacy of a two-session short-intervention using IR. Furthermore, the intervention was supported by an app-based DHI. Specifically, the presented intervention aimed to reduce NSSI and increase several aspects of emotion dysregulation and self-efficacy in adolescents who self-harm. In six out of seven patients, there was a significant reduction in NSSI behavior at post-treatment, with an average decrease of 64% compared to pre-treatment. Furthermore, there was a decrease in NSSI-related thoughts in four out of seven patients at post-treatment. In line with previous research, the current findings indicate that both emotion regulation strategies and NSSI showed concomitant improvements. This finding provides additional evidence to support the hypothesis that NSSI may be utilized as a means to regulate aversive emotions. Moreover, the changes in NSSI within the current sample are comparable to those recently reported by Schaitz et al. (2020), as well as Sosic-Vasic et al. (2024) who investigated adult patients diagnosed with BPD displaying EDB. Within the intervention group, 36% of BPD patients experienced a significant improvement in symptoms immediately after treatment, and in 28% this improvement persisted 3 months later (Schaitz et al., 2020). Examining a more extensive range of studies on psychiatric disorders treated with IR, findings reveal that IR is an effective method for ameliorating symptoms such as depression (Brewin et al., 2009), social anxiety (Wild and Clark, 2011), and PTSD (Jung and Steil, 2013; Kroener et al., 2023). Furthermore, recent research has found a linkage between NSSI and dissociative symptoms (Nester et al., 2023), which would be an interesting target for future studies investigating therapeutic treatments utilizing IR. For patients who conduct NSSI, such as the population investigated within the current study, the process of re-scripting mental images seems to facilitate a modification within the distorted emotional processing (Schaitz et al., 2020; Sosic-Vasic et al., 2024). The Emotional Cascade Model proposed by Selby Edward et al. (2008) outlines a process in which rumination and negative affect intensify emotions resulting in impulsive and maladaptive behaviors, such as NSSI. Prior studies have substantiated this hypothesis by revealing a continuous presence of NSSI-related images preceding the conduct thereof (Schaitz et al., 2020). These images are characterized by a lack of control and a high level of intrusiveness (Schaitz et al., 2020). The utilization of IR allows individuals who struggle with NSSI to analyze triggers and reinforce the process of reevaluating these kinds of situations. This, in turn, augments their self-efficacy, as they are able to exert control over their thoughts and direct their attention toward available resources. Equipping adolescent patients with methods to manage and modify these distressing mental images, such as utilizing IR strategies, has the potential to decrease overall clinical symptoms, which will be discussed in the following paragraph.

At the three-month follow-up, participants demonstrated a notable 32% increase in the utilization of reappraisal strategies, resulting in a shift in their cognitive processes and a reduction in negative emotions. These techniques were acquired during the IR sessions, within which the imagined future situation was imaginatively altered in order to achieve a more positive outcome. This aligns with the core premise of IR, which focuses on fulfilling emotional needs by transforming intrusive, negative imagery into more positive mental images (Holmes et al., 2007). However, it is imperative to take the possibility of spontaneous remission from NSSI into account, which may be attributed to variables such as brain maturation (Margit et al., 2023), external and social influences such academic distress, or familial conflicts (Tilton-Weaver et al., 2023). In order to be able to provide a more precise conclusion about treatment effects following IR as well as external factors contributing to spontaneous remission, future randomized controlled trials are needed including a larger sample size. Comparable to recent findings, the dysfunctional emotion regulation strategy suppression decreased significantly by 17% during the first month following treatment termination (Schaitz et al., 2020). Furthermore, there was a 14% trend decrease at three-months follow-up. These results are comparable to the findings provided by Schaitz et al. (2020), who conducted a study investigating patients diagnosed with Borderline Personality Disorder (BPD) engaging in NSSI. As the present study did not establish a concurrent BPD diagnosis, future research could investigate the possible beneficial effects of treating adolescents diagnosed with BPD with IR.

Trait anxiety is a relatively stable inter-individual difference in the tendency to evaluate situations as threatening. We were able to observe a symptom reduction for this parameter over time, consistent with the expected effect based on the increase in the use of the reappraisal strategy. This significant decrease of trait anxiety between T0 and FU2 revealed a medium to large effect (*d* = 1.99), which is in close agreement with the results of a meta-analysis on IR by Kip et al. (2023).

Similar to the findings in recent studies, there is a linkage between NSSI and depressive symptoms (Schaitz et al., 2020). The depressive symptoms of our patients improved with a clinical significance of 33% for up to 3 months after treatment.

Furthermore, there was an improvement of general self-efficacy within 3 months, where our observed large effect sizes (WIRKALL_r: *d* = 1.57) indicate a high degree of clinical significance.

In our study, the two sessions IR were well received and perceived as helpful and of good quality based on BIKEB results. Comparable to previous studies reporting a low dropout rate, we did not encounter any dropouts, indicating a high acceptance rate of IR techniques (Müller-Engelmann and Steil, 2017). Furthermore, most of the participants showed a high adherence to homework and the majority continued using the mental image for at least 3 months. Based on the lack of state-of-the-art therapies like DBT(Kothgassner et al., 2021), disorder specific digital interventions as our app-based DHI are a much needed tool to fill the gap between diagnosis and treatment. Particularly, considering that only 7.9% of self-harming adolescents are willing to disclose this behavior to contacted professionals (Demuthova et al., 2020), largely due to feelings of shame and guilt. The use of an app could prove beneficial, as it routinely assesses self-harming behavior, without having to disclose this behavior during a face-to-face interaction. Although most patients benefited from the intervention, two patients did not improve across all measures. Firstly, patient 5 benefited from the intervention, but her scores worsened at post-treatment. At the time of the assessment, this patient was in the middle of her finals for her high school diploma, therefore experiencing a large amount of distress. Furthermore, the treatment received within our study was the first time that this patient received care for NSSI. Speaking about her mental illness for the first time was challenging for this patient. Furthermore, she reported feeling different than her peers, which further increased her stress level. All of these factors together might have contributed to increased scores across the various scales. Secondly, patient 4 did not benefit from the treatment as indicated by increased scores of NSSI. During the diagnostic interview, the patient reported engaging in self-injurious behaviors such as biting her inner cheek and chewing her fingernails. This behavior was performed as self-injury, but also happened as an involuntary reaction to high levels of stress, without any awareness. Within the diagnostic session, the distinction between a reflex (or tic) and self-harm was discussed. During the therapeutic sessions, this patient became increasingly aware of her self-injurious behavior. Moreover, she was receiving daily reminders from the app to report any instances of NSSI. Therefore, it remains unclear whether the increase in reported NSSI behaviors and thoughts might have stemmed from an increased awareness of the occurrence thereof. This hypothesis could serve as an explanation as to why her scores on NSSI and related thoughts increased, while all other outcome measures improved, and the provided app feedback was very positive. Therefore, future studies could include measures on interoception to investigate possible moderating influences of this construct.

### 4.1 Limitations

The present study is limited in generalizability, as the study only included seven female patients suffering from NSSI. As the majority of patients presenting symptoms of NSSI are female (Sansone and Sansone, 2011), the present study nevertheless represents everyday clinical applicability. Second, symptom improvements, as described within our study, must be interpreted with caution due to the lack of a control group. Third, only two treatment sessions were conducted. It might be possible that including additional targeted intervention sessions could further increase overall treatment efficacy. Additionally, booster sessions could be offered after a set period to allow for consolidation. Moreover, we cannot preclude the possibility of non-specific treatment effects, such as the therapeutic alliance, which might have additionally contributed to symptom improvement. Taken together, this study highlights the need for future randomized controlled trials to replicate the presented preliminary results.

## 5 Conclusion

The findings of the present study indicate a good efficacy and feasibility for this newly developed sort-intervention using IR for adolescents conducting NSSI. After two-sessions of IR, symptoms of NSSI and depressiveness were reduced, while self-efficacy and emotion-focused reappraisal increased. Patients reported high treatment satisfaction and stated that the included DHI was experienced as very supportive. While our study solely consists of individual cases, it indicates that IR is a promising treatment for adolescents who self-injure. Conducting randomized controlled studies with larger sample sizes is necessary to establish reliable empirical proof for the treatment’s efficacy.

## Data availability statement

The data will be available from the corresponding author upon reasonable request.

## Ethics statement

The studies involving humans were approved by Ethikkommission Universität Ulm, Ulm. The studies were conducted in accordance with the local legislation and institutional requirements. Written informed consent for participation in this study was provided by the participants’ legal guardians/next of kin.

## Author contributions

ES: Conceptualization, Data curation, Investigation, Methodology, Software, Validation, Writing – original draft, Writing – review & editing. LH: Data curation, Writing – review & editing. BC: Resources, Writing – review & editing. ZS-V: Conceptualization, Methodology, Project administration, Resources, Supervision, Writing – review & editing. JK: Formal analysis, Supervision, Writing – review & editing, Methodology, Visualization, Writing – original draft.

## References

[ref1] Abler B. Kessler H. (2009). Emotion Regulation Questionnaire–Eine deutschsprachige Fassung des ERQ von Gross und John. Diagnostica 55, 144–152. doi: 10.1026/0012-1924.55.3.144

[ref2] Andover M. S. Morris B. W. (2014). Expanding and clarifying the role of emotion regulation in nonsuicidal self-injury. Can. J. Psychiatr. 59, 569–575. doi: 10.1177/070674371405901102, PMID: 25565472 PMC4244875

[ref3] Association, American Psychiatric (2013). Diagnostic and statistical manual of mental disorders. US: American Psychiatric Association.

[ref4] Brewin C. R. Wheatley J. Patel T. Fearon P. Hackmann A. Wells A.. (2009). Imagery rescripting as a brief stand-alone treatment for depressed patients with intrusive memories. Behav. Res. Ther. 47, 569–576. doi: 10.1016/j.brat.2009.03.008, PMID: 19345934

[ref5] Czyz E. K. Horwitz A. G. Eisenberg D. Kramer A. King C. A. (2013). Self-reported barriers to professional help seeking among college students at elevated risk for suicide. J. American college health: J of ACH 61, 398–406. doi: 10.1080/07448481.2013.820731, PMID: 24010494 PMC3788673

[ref6] Demuthova S. Vaclavikova I. Selecka L. Blatny M. (2020). The problem of self-disclosure of self-harming behaviour in adolescence. PO 11, 01–19. doi: 10.18662/po/11.4/220

[ref7] Esposito C. Dragone M. Affuso G. Amodeo A. L. Bacchini D. (2023). Prevalence of engagement and frequency of non-suicidal self-injury behaviors in adolescence: an investigation of the longitudinal course and the role of temperamental effortful control. Eur. Child Adolesc. Psychiatry 32, 2399–2414. doi: 10.1007/s00787-022-02083-7, PMID: 36123505 PMC10682258

[ref8] Field Andy P. (2013). Discovering statistics using IBM SPSS statistics. 4th ed. London, Sage Publications.

[ref9] Grimm J. (2009). State-Trait-Anxiety Inventory nach Spielberger. Deutsche Lang-und Kurzversion Methodenforum der Universität Wien: MF-Working Paper 2009/02.

[ref10] Groschwitz R. C. Plener P. L. Kaess M. Schumacher T. Stoehr R. Boege I. (2015). The situation of former adolescent self-injurers as young adults: a follow-up study. BMC Psychiatry 15:160. doi: 10.1186/s12888-015-0555-1, PMID: 26187150 PMC4506399

[ref11] Hackmann A. Clark D. M. McManus F. (2000). Recurrent images and early memories in social phobia. Behav. Res. Ther. 38, 601–610. doi: 10.1016/s0005-7967(99)00161-8, PMID: 10846808

[ref12] Hasking P. A. Simplicio D. McEvoy M. Peter M. Rees C. S. (2018). Emotional cascade theory and non-suicidal self-injury: the importance of imagery and positive affect. Cognit. Emot. 32, 941–952. doi: 10.1080/02699931.2017.1368456, PMID: 28838289 PMC6050645

[ref13] Hautzinger M. Keller F. Kühner C. (2006). BDI-II. Beck depressions inventar revision—Manual. Frankfurt, Harcourt Test Services.

[ref14] Hawton K. Bergen H. Cooper J. Turnbull P. Waters K. Ness J.. (2015). Suicide following self-harm: findings from the multicentre study of self-harm in England, 2000-2012. J. Affect. Disord. 175, 147–151. doi: 10.1016/j.jad.2014.12.062, PMID: 25617686

[ref15] Höger D. Eckert J. (1997). Der Bielefelder Klienten-Erfahrungsbogen (BIKEB). Ein Verfahren zur Erfassung von Aspekten des “Post-Session Outcome” bei Psychotherapien. Z. Klin. Psychol. 26, 129–137.

[ref16] Hollis C. Falconer C. J. Martin J. L. Whittington C. Stockton S. Glazebrook C.. (2017). Annual research review: digital health interventions for children and young people with mental health problems - a systematic and meta-review. J. Child Psychol. Psychiatry 58, 474–503. doi: 10.1111/jcpp.12663 27943285

[ref17] Holmes E. A. Arntz A. Smucker M. R. (2007). Imagery rescripting in cognitive behaviour therapy: images, treatment techniques and outcomes. J. Behav. Ther. Exp. Psychiatry 38, 297–305. doi: 10.1016/j.jbtep.2007.10.007, PMID: 18035331

[ref18] Holmes E. A. Ghaderi A. Harmer C. J. Ramchandani P. G. Cuijpers P. Morrison A. P.. (2018). The lancet psychiatry commission on psychological treatments research in tomorrow's science. Lancet Psychiatry 5, 237–286. doi: 10.1016/s2215-0366(17)30513-8, PMID: 29482764

[ref19] Holmes E. A. Mathews A. (2005). Mental imagery and emotion: a special relationship? Emotion 5, 489–497. doi: 10.1037/1528-3542.5.4.489 16366752

[ref20] Holmes E. A. Mathews A. (2010). Mental imagery in emotion and emotional disorders. Clin. Psychol. Rev. 30, 349–362. doi: 10.1016/j.cpr.2010.01.001 20116915

[ref21] Jacobson N. S. Truax P. (1991). Clinical significance: a statistical approach to defining meaningful change in psychotherapy research. J. Consult. Clin. Psychol. 59, 12–19. doi: 10.1037/0022-006x.59.1.12, PMID: 2002127

[ref22] James A. C. Taylor A. Winmill L. Alfoadari K. (2008). A preliminary community study of dialectical behaviour therapy (DBT) with adolescent females demonstrating persistent, deliberate self-harm (DSH). Child Adolesc. Mental Health 13, 148–152. doi: 10.1111/j.1475-3588.2007.00470.x, PMID: 32847177

[ref23] Jung K. Steil R. (2013). A randomized controlled trial on cognitive restructuring and imagery modification to reduce the feeling of being contaminated in adult survivors of childhood sexual abuse suffering from posttraumatic stress disorder. Psychother. Psychosom. 82, 213–220. doi: 10.1159/000348450, PMID: 23712073

[ref24] Kip A. Schoppe L. Arntz A. Morina N. (2023). Efficacy of imagery rescripting in treating mental disorders associated with aversive memories - an updated meta-analysis. J. Anxiety Disord. 99:102772. doi: 10.1016/j.janxdis.2023.102772, PMID: 37699277

[ref25] Knäuper B. Roseman M. Johnson P. J. Krantz L. H. (2009). Using mental imagery to enhance the effectiveness of implementation intentions. Curr. Psychol. 28, 181–186. doi: 10.1007/s12144-009-9055-0

[ref26] Kothgassner O. D. Goreis A. Robinson K. Huscsava M. M. Schmahl C. Plener P. L. (2021). Efficacy of dialectical behavior therapy for adolescent self-harm and suicidal ideation: a systematic review and meta-analysis. Psychol. Med. 51, 1057–1067. doi: 10.1017/s0033291721001355, PMID: 33875025 PMC8188531

[ref27] Kothgassner O. D. Robinson K. Goreis A. Ougrin D. Plener P. L. (2020). Does treatment method matter? A meta-analysis of the past 20 years of research on therapeutic interventions for self-harm and suicidal ideation in adolescents. Borderline personality disorder and emotion dysregulation 7:9. doi: 10.1186/s40479-020-00123-9 32426138 PMC7216729

[ref28] Kroener J. Hack L. Mayer B. Sosic-Vasic Z. (2023). Imagery rescripting as a short intervention for symptoms associated with mental images in clinical disorders: a systematic review and meta-analysis. J. Psychiatr. Res. 166, 49–60. doi: 10.1016/j.jpsychires.2023.09.010 37738780

[ref29] Larsen M. E. Nicholas J. Christensen H. (2016). A systematic assessment of smartphone tools for suicide prevention. PLoS One 11:e0152285. doi: 10.1371/journal.pone.0152285, PMID: 27073900 PMC4830444

[ref30] Lee M. Lee H. Kim Y. Kim J. Cho M.. (2018). Mobile app-based health promotion programs: a systematic review of the literature. Int. J. Environ. Res. Public Health 15:2838. doi: 10.3390/ijerph15122838, PMID: 30551555 PMC6313530

[ref31] Libby L. K. Shaeffer E. M. Eibach R. P. Slemmer J. A. (2007). Picture yourself at the polls: visual perspective in mental imagery affects self-perception and behavior. Psychol. Sci. 18, 199–203. doi: 10.1111/j.1467-9280.2007.01872.x 17444910

[ref32] Margit W.-L. Lars-Gunnar L. Benjamin C. Jonas B. Daiva D. (2023). Developmental pathways of repetitive non-suicidal self-injury: predictors in adolescence and psychological outcomes in young adulthood. Child Adolesc. Psychiatry Ment. Health 17:S116. doi: 10.1186/s13034-023-00660-5 PMC10571303 37833783

[ref33] May J. Andrade J. Kavanagh D. J. (2015). An imagery-based road map to tackle maladaptive motivation in clinical disorders. Front. Psychol. 6:14. doi: 10.3389/fpsyt.2015.00014, PMID: 25729369 PMC4325580

[ref34] McEvoy P. M. Hayes S. Hasking P. A. Rees C. S. (2017). Thoughts, images, and appraisals associated with acting and not acting on the urge to self-injure. J. Behav. Ther. Exp. Psychiatry 57, 163–171. doi: 10.1016/j.jbtep.2017.05.010 28601695

[ref36] Müller-Engelmann M. Steil R. (2017). Cognitive restructuring and imagery modification for posttraumatic stress disorder (CRIM-PTSD): a pilot study. J. Behav. Ther. Exp. Psychiatry 54, 44–50. doi: 10.1016/j.jbtep.2016.06.004, PMID: 27344103

[ref1002] Nester M. S. Pierorazio N. A. Shandler G. Brand B. L. (2023). Characteristics, methods, and functions of non-suicidal self-injury among highly dissociative individuals. J. Trauma Dissoc. 24, 333–47. 10.1080/15299732.2023.2181475 36803534

[ref37] Nock M. K. Joiner T. E. Gordon K. H. Lloyd-Richardson E. Prinstein M. J. (2006). Non-suicidal self-injury among adolescents: diagnostic correlates and relation to suicide attempts. Psychiatry Res. 144, 65–72. doi: 10.1016/j.psychres.2006.05.010 16887199

[ref38] Ofcom (2015). Children and parents: media use and Attidude report. Int. communications market report. Available at: https://www.ofcom.org.uk/__data/assets/pdf_file/0024/78513/childrens_parents_nov2015.pdf

[ref39] Piccirillo M. L. Burke T. A. Moore-Berg S. L. Alloy L. B. Heimberg R. G. (2020). Self-stigma toward nonsuicidal self-injury: an examination of implicit and explicit attitudes. Suicide Life Threat. Behav. 50, 1007–1024. doi: 10.1111/sltb.12640, PMID: 32462657 PMC11137797

[ref40] Plener P. L. Allroggen M. Kapusta N. D. Brähler E. Fegert J. M. Groschwitz R. C. (2016). The prevalence of nonsuicidal self-injury (NSSI) in a representative sample of the German population. BMC Psychiatry 16:353. doi: 10.1186/s12888-016-1060-x, PMID: 27760537 PMC5069807

[ref41] Remschmidt H. Schmidt M. H. Poustka F. (2017). “Multiaxiales Klassifikationsschema für psychische Störungen des Kindes-und Jugendalters nach ICD-10” in Mit einem synoptischen Vergleich von ICD-10 und DSM-5. 7. aktualisierte Auflage. eds. Remschmidt H. Schmidt M. H. Poustka F. (Bern: Hogrefe (Programmbereich Psychiatrie))

[ref42] Rowe S. L. French R. S. Henderson C. Ougrin D. Slade M. Moran P. (2014). Help-seeking behaviour and adolescent self-harm: a systematic review. Aust. N. Z. J. Psychiatry 48, 1083–1095. doi: 10.1177/0004867414555718, PMID: 25335872

[ref43] Sansone R. A. Sansone L. A. (2011). Gender patterns in borderline personality disorder. Innovations in clinical neuroscience 8, 16–20. PMID: 21686143 PMC3115767

[ref44] Schaitz C. Kroener J. Maier A. Connemann B. J. Sosic-Vasic Z. (2020). Short imagery Rescripting intervention to treat emotionally dysregulated behavior in borderline personality disorder: an exploratory study. Front. Psychol. 11:425. doi: 10.3389/fpsyt.2020.00425, PMID: 32508686 PMC7251139

[ref46] Schwarzer R. (1999). “Skalen zur Erfassung von Lehrer-und Schülermerkmalen” in Dokumentation der psychometrischen Verfahren im Rahmen der wissenschaftlichen Begleitung des Modellversuchs Selbstwirksame Schulen (Berlin: R. Schwarzer)

[ref47] Selby Edward A. Anestis Michael D. Joiner Thomas E. (2008). Understanding the relationship between emotional and behavioral dysregulation: emotional cascades. Behav. Res. Ther. 46, 593–611. doi: 10.1016/j.brat.2008.02.002, PMID: 18353278

[ref48] Sheehan D. V. Lecrubier Y. Sheehan K. H. Amorim P. Janavs J. Weiller E.. (1998). The Mini-international neuropsychiatric interview (M.I.N.I.): the development and validation of a structured diagnostic psychiatric interview for DSM-IV and ICD-10. J. Clin. Psychiatry 59, 22–33. PMID: 9881538

[ref49] Simplicio D. Appiah-Kusi M. Wilkinson E. Watson P. Meiser-Stedman P. Kavanagh C.. (2020). Imaginator: a proof-of-concept feasibility trial of a brief imagery-based psychological intervention for young people who self-harm. Suicide Life Threat. Behav. 50, 724–740. doi: 10.1111/sltb.12620, PMID: 32057131 PMC7613067

[ref50] Sosic-Vasic Z. Schaitz C. Mayer B. Maier A. Connemann B. Kroener J. (2024). Treating emotion dysregulation in patients with borderline personality disorder using imagery rescripting: a two-session randomized controlled trial. Behav. Res. Ther. 173:104454. doi: 10.1016/j.brat.2023.104454, PMID: 38194759

[ref51] Tilton-Weaver L. Latina D. Marshall S. K. (2023). Trajectories of nonsuicidal self-injury during adolescence. J. Adolesc. 95, 437–453. doi: 10.1002/jad.12126 36437557

[ref52] Torous J. Friedman R. Keshavan M. (2014). Smartphone ownership and interest in mobile applications to monitor symptoms of mental health conditions. JMIR Mhealth Uhealth 2:e2. doi: 10.2196/mhealth.2994, PMID: 25098314 PMC4114412

[ref53] Wild J. Clark D. M. (2011). Imagery Rescripting of early traumatic memories in social phobia. Cogn. Behav. Pract. 18, 433–443. doi: 10.1016/j.cbpra.2011.03.002, PMID: 22298942 PMC3267018

